# Serine protease PRSS23 drives gastric cancer by enhancing tumor associated macrophage infiltration *via* FGF2

**DOI:** 10.3389/fimmu.2022.955841

**Published:** 2022-09-15

**Authors:** Shanshan Qin, Zidi Wang, Congcong Huang, Pan Huang, Dandan Li

**Affiliations:** ^1^ Hubei Key Laboratory of Embryonic Stem Cell Research, School of Basic Medical Sciences, Hubei University of Medicine, Shiyan, China; ^2^ Laboratory of Tumor Biology, Academy of Bio-Medicine Research, Hubei University of Medicine, Shiyan, China

**Keywords:** serine protease PRSS23, FGF2, macrophage infiltration, TAM, gastric cancer

## Abstract

Serine proteases has been considered to be closely associated with the inflammatory response and tumor progression. As a novel serine protease, the biological function of PRSS23 is rarely studied in cancers. In this study, the prognostic significance of PRSS23 was analyzed in two-independent gastric cancer (GC) cohorts. PRSS23 overexpression was clinically correlated with poor prognosis and macrophage infiltration of GC patients. Loss-of-function study verified that PRSS23 plays oncogenic role in GC. RNA-seq, qRT-PCR, western blotting and ELISA assay confirmed that serine protease PRSS23 positively regulated FGF2 expression and secretion. Single-cell analysis and gene expression correlation analysis showed that PRSS23 and FGF2 were high expressed in fibroblasts, and highly co-expressed with the biomarkers of tumor associated macrophages (TAMs), cancer-associated fibroblasts (CAFs) and mesenchymal cells. Functional analysis confirmed PRSS23/FGF2 was required for TAM infiltration. Rescue assay further verified that PRSS23 promotes GC progression and TAM infiltration through FGF2. Survival analysis showed that high infiltration of M1-macrophage predicted favorable prognosis, while high infiltration level of M2-macrophage predicted poor prognosis in GC. Our finding highlights that PRSS23 promotes TAM infiltration through regulating FGF2 expression and secretion, thereby resulting in a poor prognosis.

## Introduction

Gastric cancer (GC) is a heterogeneous tumor with the third highest mortality rate worldwide (1). There are about 1.089 million new cases of gastric cancer worldwide in 2020, of which about 478,508 cases occurred in China (2, 3). Though current treatments for patients have been greatly improved, the prognosis remains unoptimistic to date due to the inconvenience of early diagnosis of GC (4). Besides, the molecular mechanisms underlying GC progression remain unclear (5–8). Hence, it is urgent and necessary to explore novel potential biomarkers and their molecular mechanisms to better understand the pathophysiology of gastric malignancies.

Serine proteases play critical roles in the digestion, blood coagulation fertilization, fibrinolysis, cell apoptosis and differentiation, angiogenesis (9). Recently, emerging evidence have showed that serine proteases play essential roles in tumor progression. For examples, Serine protease PRSS8 suppresses colorectal carcinogenesis and metastasis by inhibiting epithelial mesenchymal transition (EMT) signaling (10, 11). Serine protease PRSS3 was found to function as an oncogene in stomach cancer, lung cancer and colon cancer (12–14). However, as a conserved member of the trypsin family of serine proteases (15), the biological function of serine protease PRSS23 remains largely unknown in cancers.

Tumor-associated macrophages (TAMs) have been reported to be independent prognostic biomarker in cancers, including GC (16–18). Increasing studies have reported that TAMs exert pro-tumor effects by inhibiting antitumor immune responses (19). TAMs closely resemble the M2-macrophages, both of which highly express classic biomarkers of M2 macrophage, such as CD163, MSR1, and MRC1 (20–22). Fibroblast growth factor 2 (FGF2), secreted by cancer-associated fibroblast (CAFs), was reported to be required for tumor cell growth in lung cancer (23). Recently, multiple independent studies have reported a critical role of FGF2 in TAM infiltration, which implied a pro-tumor role of FGF2 in tumor progression (24–26).

In this study, a novel role of serine protease PRSS23 in immune infiltration was disclosed in GC. PRSS23 overexpression was positively associated with poor prognosis and macrophage infiltration in GC. PRSS23 functions as an oncogene in GC by enhancing tumor associated macrophage infiltration *via* FGF2. Our data highlights that the upregulation of PRSS23/FGF2 may be critical for macrophage infiltration in pan-cancer.

## Materials and methods

### Prognostic analysis and single-cell analysis

The gene expression profile of GSE62254 used in this study was downloaded from the Gene Expression Omnibus (GEO) in the NCBI web server. The clinical information of GC patients from GSE62254 cohort was download as descripted previously (27). The gene expression data and the clinical information of GC patients were obtained from the Cancer Genome Atlas (TCGA) database. Expression level of per gene was calculated from log2 of FPKM-UQ value. Single-cell analysis used in this study was obtained from the Human Protein Atlas (HPA) dataset (https://www.proteinatlas.org/).

### Immune infiltration analysis

The TIMER database can used to estimate the immune infiltration levels of B cells, CD4+ T cells, CD8+ T cells, Neutrophils, Macrophages and Dendritic cells. The CIBERSORT method can used to estimate the immune infiltration of 24 immune cell types. The quanTIseq method can used to estimate the immune infiltration of 10 immune cell types, including M1 and M2 macrophages. These algorithms provide powerful correlation analysis and survival analysis regarding different types of immune cells. The gene module allows users to select any gene of interest and visualize the correlation of its expression with immune infiltration level in diverse cancer types. The survival module allows users to explore the clinical relevance of one or more tumor immune subsets, with the flexibility to correct for multiple covariates in a multivariable Cox proportional hazard model. The gene expression level in different immune cell types between stomach cancer and normal stomach tissues was analyzed using GEPIA 2021 web tool.

### Cell culture and cell transfection

For cell culture, all cell lines used in this study were cultured in DMEM medium containing 10% fetal bovine serum (FBS) at 37 °C in 5% CO_2_. The siRNAs targeting PRSS23 were purchased from Genepharma (Shanghai, China). The sequence of 2 siRNAs targeting PRSS23 were listed as follows. siRNA#1: 5’-GCGGCAGAUUUAUGGCUAUTT-3’, siRNA#2: 5’-CCAGAUUUGCUAUUGGAUUTT-3’. For cell transfection, the GC cells were plated into a six-well plate. After the cell density reaches 30-50% the next day, siRNAs were transfected into GC cells using Lipofectamine 2000 (Invitrogen) according to the manufacturer’s instructions.

### THP-1-derived TAMs

THP-1 cells were used to induce TAMs *in vitro* as described previously (28–30). Briefly, macrophages were induced from THP-1 cells by treatment with PMA (Sigma, 100 ng/mL) for 24 hours. Then, these THP-1 derived macrophages were re-placed into a six-well transwell plate. At the same time, HGC-27 cells were cultured as usually on the 0.4-μm porous membrane of upper chamber. After 24 hours, we co-cultured HGC-27 cells with THP-1-derived macrophages. Then 48 hours later, macrophages were collected for RNA extraction and other experiments.

### Quantitative RT-PCR assay

At 48 hours post-transfection, GC cells were directly harvested using Trizol reagent (Invitrogen, USA) and the total RNA was extracted according to the manufacturer’s instructions. The contaminated gDNA in total RNA was removed using RNase-free DNase I (Roche) for 20 minutes (31). cDNA was obtained using the PrimeScript™ RT reagent Kit (Perfect Real Time, Takara). The qPCR analysis was performed on Bio-Rad CFX Manager 3.1 real-time PCR system. The specific primers used in this study were synthesized by Wcgene Biotech (Shanghai, China). FGF2-F: 5’-GAAAAGGCAAGATGCAGGAG-3’, FGF2-R: 5’-ACGTGAGAGCAGAGCATGTG-3’; PRSS23-F: 5’-GGGGGATTTTCTGCTTGTCT-3’, PRSS23-R: 5’- TGGAGACCTCCCTTCTTCCT-3’; ACTIN-F: 5’-ATCGTCCACCGCAAATGCTTCTA-3’, ACTIN-R: 5’-AGCCATGCCAATCTCATCTTGTT-3’ 2 ^–∆∆Ct^ method was used to determine gene expression quantification.

### Western blotting assay

The western blotting assay was performed as previously described (1). In brief, after 72h transfected with siRNAs, GC cells were lysed in RIPA buffer added 1 mM PMSF. Approximately 100 μg of total protein was electrophoresed through 10% SDS polyacrylamide gels and were then transferred to a PVDF membrane (Millipone). The FGF2 antibody (A11488) and PRSS23 (A17092) antibody was purchased from Abclonal company (Wuhan, China).

### RNA sequencing

After transfection of 2 siRNAs targeting PRSS23 in AGS cells, total RNA was extracted and send to Lifegenes company (Shanghai, China) to perform RNA sequencing. A total amount of 1.5 µg RNA per sample was used as input material for the RNA sample preparations. The RNA-seq data used in this study was uploaded in the GEO dataset (GSE204725).

### Statistical analysis

The P values for PRSS23 expression analysis of different subtypes of GC were estimated using Mann–Whitney nonparametric test. The P values of survival curves were analyzed using the log-rank test. Pearson correlation analysis was used for the correlation test of the two groups of data. For quantitative RT-PCR, the P values were analyzed using ANOVA. P < 0.05 considered statistically significant.

## Results

### Serine protease PRSS23 overexpression predicts poor prognosis in GC

**Figure 1:**
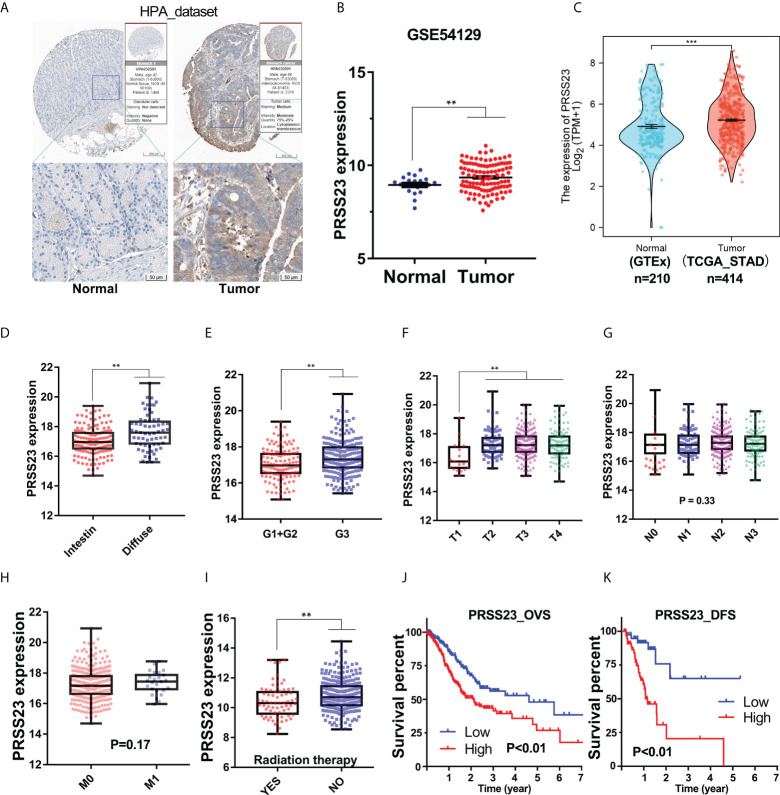
The clinical significance of PRSS23 overexpression was analyzed in the GC cohort from TCGA. **(A)** Differences in the immunostaining of PRSS23 between normal tissues and cancerous tissues in GC. **(B, C)** PRSS23 was overexpressed in cancerous tissues in the GSE54129 and TCGA_STAD cohort. **(D)** Differences in PRSS23 expression between intestinal and diffuse tissues of GC. **(E)** PRSS23 expression in GC tissues with different differentiation stages. **(F–H)** PRSS23 expression level in different TNM-stages of GC tissues. **(I)** PRSS23 was lowly expressed in GC patients with radiation therapy. **(J, K)**: PRSS23 overexpression predicted shorter overall survival time and disease-free survival time in GC. **, P < 0.01, ***, P < 0.001.

To reveal the biological function of PRSS23 in GC, we firstly analyzed its expression pattern in GC and normal stomach tissues. The Human Protein Atlas (HPA) contains large quantity of immunohistochemistry (IHC) images of different proteins in normal human tissues and cancer tissues (32). Therefore, we first evaluated the protein expression of PRSS23 in normal and cancer tissue of stomach using the HPA web tool (**Figure 1A**). The results showed that PRSS23 protein was mainly located in cytoplasmic and was relatively highly expressed in GC tissue compared to the normal stomach tissue. In addition, two independent GC cohort (GSE54129 and TCGA_STAD) containing normal tissues and cancer tissues were included into our study. The results showed that PRSS23 expression was also significantly upregulated in the GSE54129 cohort (**Figures 1B, C**).

To understand the significance of PRSS23 overexpression in GC, we analyzed the prognostic value of PRSS23 in two independent GC cohort (TCGA_STAD and GSE62254). In the TCGA_STAD cohort, PRSS23 expression in diffuse GC tissues was higher than that in intestinal GC tissues (**Figure 1D**). Poorly differentiated GC tissues tended to have relatively high expression of PRSS23 (**Figure 1E**). Furthermore, PRSS23 expression level was positively correlated to T stages of GC patients (**Figure 1F**). However, there was no significant difference in the expression of PRSS23 in GC tissues with or without lymph node metastasis or distant metastasis (**Figures 1G, H**). In addition, we also noted that PRSS23 expression was significantly decreased in the GC patients with radiation therapy, compared to the GC patients without radiation therapy (**Figure 1I**). Survival analysis showed that PRSS23 overexpression predicted poor prognosis (**Figures 1J, K**).

**Figure 2:**
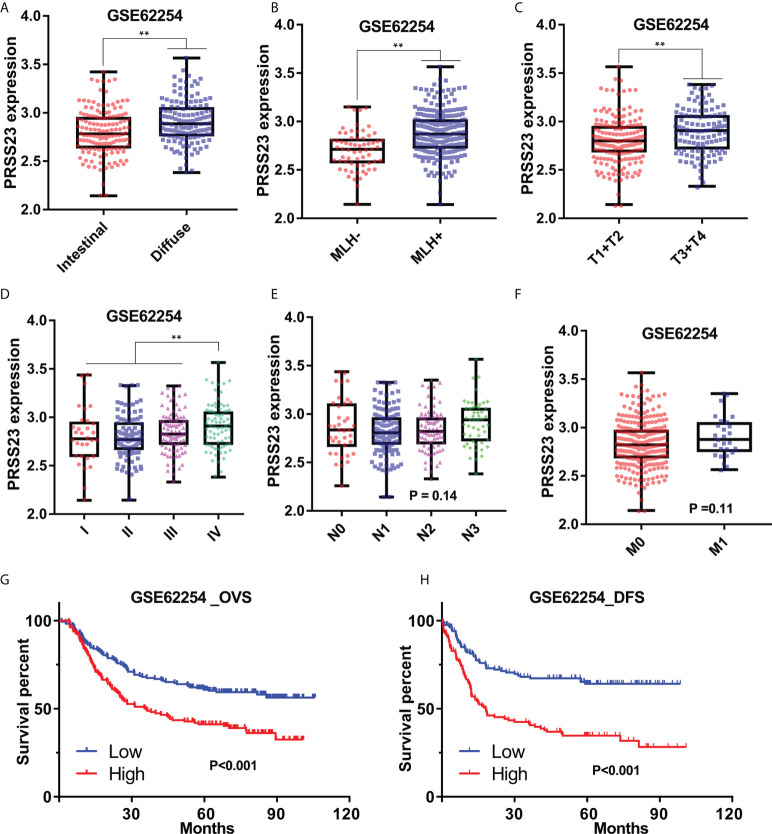
The prognostic significance of PRSS23 overexpression was analyzed in the GC cohort from GSE62254. **(A)** Differences in PRSS23 expression between intestinal and diffuse tissues of GC. **(B)** PRSS23 was highly expressed in GC patients with positive MLH1 expression. **(C–F)** PRSS23 expression level in different TNM-stages and Pathologic stages of GC tissues. **(G, H)** PRSS23 overexpression predicted shorter overall survival time and disease-free survival time in GC. **, P < 0.01.

Similarly, in GSE62254 cohort, PRSS23 was also relatively high expressed in the diffuse or MLH1+ GC tissues (**Figures 2A, B**). Furthermore, PRSS23 was positively correlated with the degree of malignancy in GC (**Figures 2C, D**), but has no significant changes in GC patients with different N/M stages (**Figures 2E, F**). Survival analysis in GSE62254 cohort also showed that PRSS23 predicted poor prognosis in GC (**Figures 2G, H**). Taken together, PRSS23 functions as an oncogene and can be served as a prognostic biomarker in GC.

### PRSS23 knockdown inhibits GC cell proliferation and invasion

**Figure 3:**
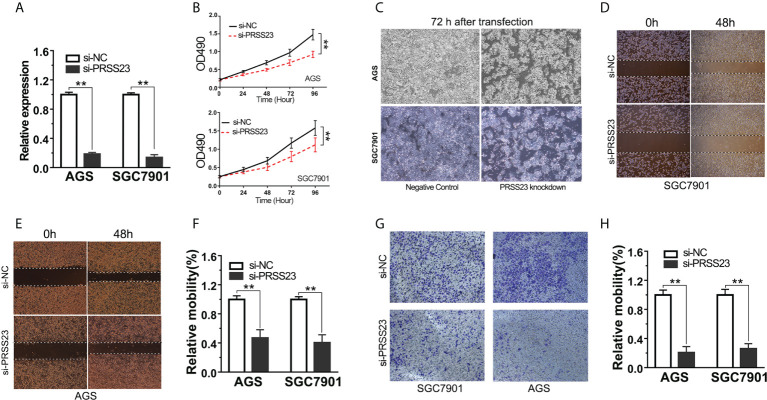
PRSS23 knockdown significantly decreased GC cell proliferation and invasion. **(A)** The efficiency of PRSS23 knockdown was determined in GC cell lines. **(B)** The effect of PRSS23 knockdown on GC cell growth was determined by MTT assay. **(C)** The morphology of gastric cancer cells after knockdown of PRSS23 for 72 hours. **(D, E)** Wound healing assays showed that PRSS23 knockdown inhibits GC cells migration. **(F)** The statistical data of the migrated cells. **(G)** The effects of PRSS23 knockdown on GC cells invasion were assessed by transwell assays. **(H)** The statistical data of the invasive cells. **, P < 0.01.

Since clinical analysis implied an oncogenic role of PRSS23 in GC, we further validated the biological function of PRSS23 *in vitro*. Given PRSS23 was overexpressed in GC tissues, we hence considered performing loss-of-function study to verify the biological function of PRSS23 in GC. Firstly, we verified the RNA interference efficiency of PRSS23 depletion in GC cell lines by qPCR assay (**Figure 3A**). Next, the cell proliferation assay showed that PRSS23 depletion caused a strong inhibition of cell growth (**Figure 3B**). After knocking down PRSS23 expression for 72 hours in GC cell lines, we checked the cell morphology with an optical microscope. The results showed that PRSS23 knockdown significantly decreased the proliferation of GC cells (**Figure 3C**). At the same time, we also determined the effect of PRSS23 knockdown on the metastasis of GC cells. In the scratch wound healing assays, the migration of GC cells that silenced PRSS23 was significantly slower than that of control GC cells (**Figures 3D–F**). In transwell invasion assays, the numbers of GC cells that invaded through the Matrigel were decreased in the PRSS23 silencing group than the control group (**Figures 3G, H**). These data demonstrated a tumor-promoting role of PRSS23 in GC.

### PRSS23 is positively associated with macrophage infiltration

**Figure 4:**
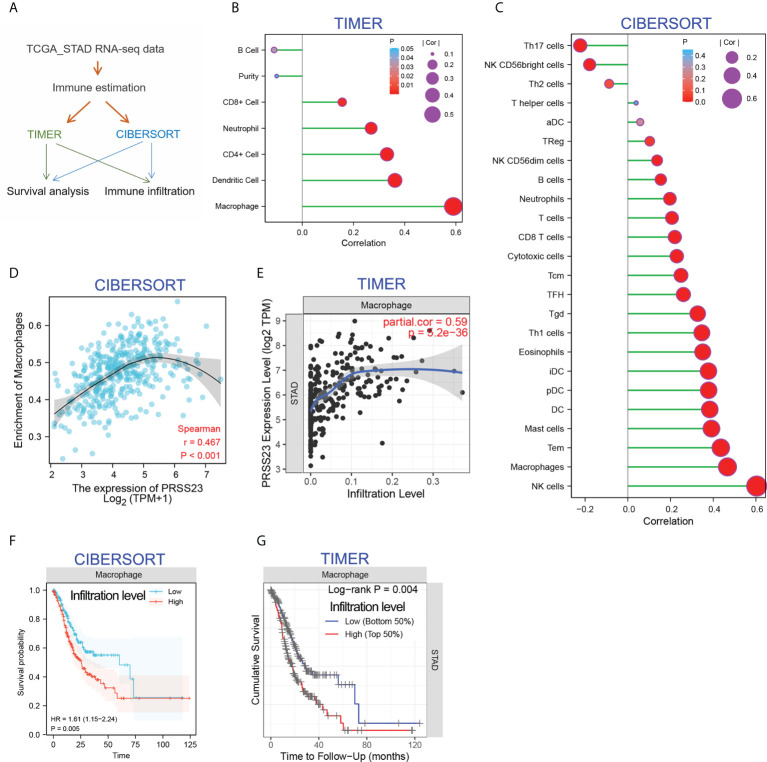
PRSS23 is associated with macrophage infiltration in GC. **(A)** Immune estimation analysis was conducted using two different methods. **(B, C)** The correlation between PRSS23 expression and immune infiltration was analyzed using TIMER and CIBERSORT methods. **(D, E)** The correlation between PRSS23 expression and macrophage infiltration was analyzed using TIMER and CIBERSORT methods. **(F**, **G)** Survival analysis using CIBERSORT or TIMER indicated that higher level of macrophage infiltration predicted poorer prognosis in GC.

Increasing studies have reported that immunity infiltration level is an independent predictor of survival and sentinel lymph node status in cancers (33). In order to clarify the biological role PRSS23 in immune infiltration, two different algorithms, including TIMER (34) and CIBERSORT (35), were performed to analyze the RNA-seq data of GC samples from TCGA (**Figure 4A**). The TIMER method contains 6 immune cell types and the CIBERSORT method contains 24 immune cell types. The infiltration level of each immune cells was evaluated by the enrichment score calculated by TIMER and CIBERSORT. Then, the correlation between PRSS23 expression level and infiltration level of each immune cell was analyzed in GC. According to immune infiltration analysis by TIMER, PRSS23 was most associated with macrophage infiltration (**Figure 4B**). Likewise, immune infiltration analysis by CIBERSORT showed that PRSS23 was most associated with macrophage and NK cell infiltration (**Figure 4C**). Scatter plots for the correlation between PRSS23 and macrophage infiltration based on two algorithms are shown in **Figures 4D, E** respectively.

Interestingly, after adjusting the clinical factors, both of the two algorithms indicated that GC patients with higher level of Macrophage infiltration tends to possess a shorter overall survival time (**Figures 4F, G**). These results suggested that PRSS23 may promote GC by affecting macrophage infiltration.

### PRSS23 knockdown decreased the expression level of FGF2 in GC

**Figure 5:**
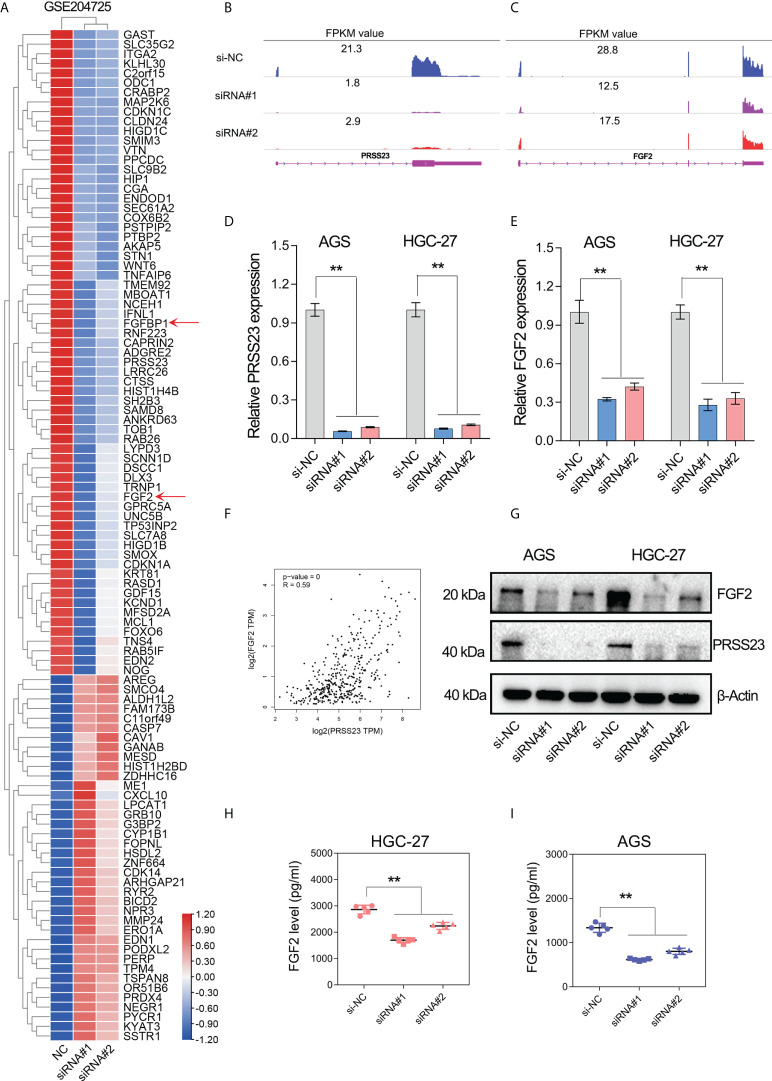
PRSS23 knockdown decreased FGF2 expression and secretion in GC. **(A)** RNA-seq studies were conducted in GC cells transfected with siRNAs targeting PRSS23. The most significantly altered genes upon PRSS23 knockdown were shown in the heatmap. **(B, C)** The transcripts abundance of PRSS23 and FGF2 in PRSS23-depleted GC cells was detected by RNA-seq. The normalized expression (FPKM value) of PRSS23 and FGF2 were shown in the plot. **(D)** The knockdown efficiency of PRSS23 in GC cell lines was examined by qRT-PCR assay. **(E)** The effect of PRSS23 knockdown on FGF2 expression in GC cell lines were examined by qRT-PCR assay. **(F)** PRSS23 and FGF2 were highly co-expressed in GC. **(G)** The effect of PRSS23 knockdown on FGF2 protein level in GC cell lines were examined by western blotting assay. (**H, I**) ELISA assay showed that PRSS23 knockdown significantly decreased secreted FGF2 level in GC cell lines. **, P < 0.01.

To figure out the molecular mechanism of PRSS23 in macrophage infiltration and GC progression, we conducted transcriptome sequencing studies (GSE204725) in GC cells between PRSS23-depleted group and control group. After analysis of the RNA-seq data, genes with the most significant fold change in expression (log2FC>0.8) after PRSS23 knockdown are listed in the heatmap (**Figure 5A**). A total of 67 genes were downregulated and 38 genes were upregulated after knockdown of PRSS23 in GC. RNA-seq analysis revealed that FGF2, which is involved in regulating macrophage polarization, was greatly decreased after PRSS23 knockdown. In addition, fibroblast growth factor-binding protein (FGFBP1), which was reported to play essential roles in regulating FGF2 secretion (36–38), was also greatly decreased after knockdown of PRSS23. Thus, we speculated that PRSS23 might regulate TAMs infiltration by regulate FGF2 secretion.

Multiple independent experiments were performed to validate the regulation of FGF2 by PRSS23 in GC. First, RNA-seq data showed that the expression of PRSS23 and FGF2 were both decreased in PRSS23-depleted GC cells (**Figures 5B, C**). Consistently, the qRT-PCR assay further confirmed that PRSS23 knockdown decreased the FGF2 expression in two GC cell lines (**Figures 5D, E**). Besides, gene expression correlation analysis also showed that PRSS23 and FGF2 were highly co-expressed in GC tissues from TCGA (**Figure 5F**). Furthermore, PRSS23 knockdown greatly reduced the protein level of FGF2 (**Figure 5G**). Given FGF2 was a secreted protein, we also examined the effect of PRSS23 knockdown on FGF2 secretion by ELISA. The ELISA assay showed that PRSS23 knockdown significantly hindered secreted FGF2 level (**Figures 5H, I**).

### PRSS23/FGF2 axis positively regulates tumor associated macrophage infiltration

**Figure 6:**
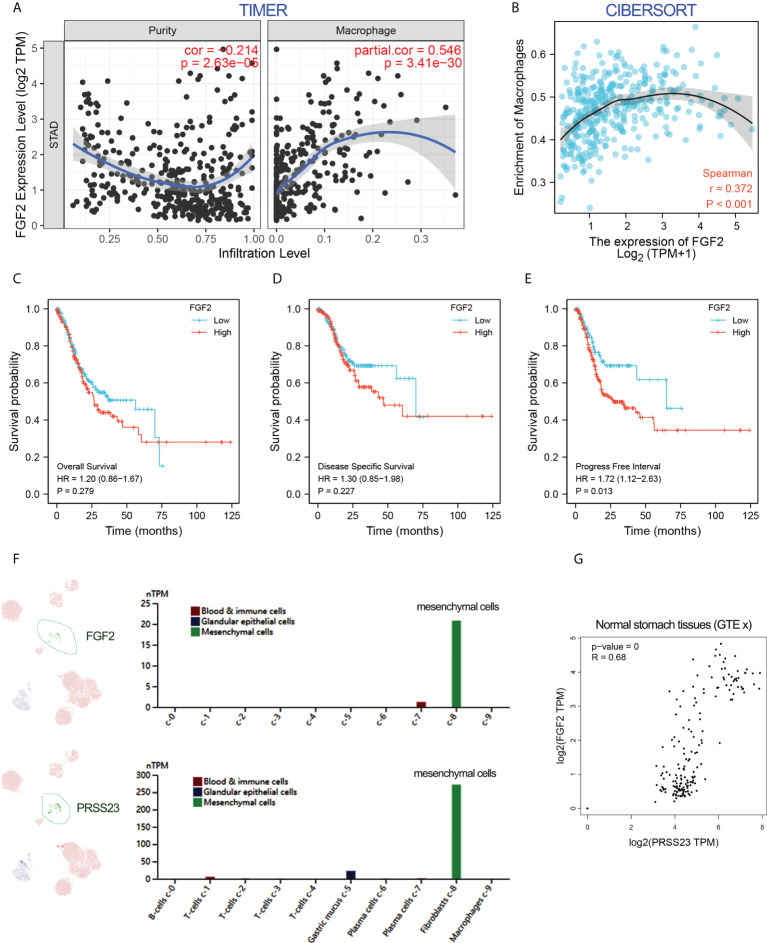
FGF2 showed a positive association with macrophage infiltration and PRSS23 expression. **(A, B)** The correlation between FGF2 expression and immune infiltration was analyzed using TIMER and CIBERSORT methods. **(C–E)** FGF2 overexpression predicted poor overall survival, disease-specific survival and progress-free survival in GC from TCGA dataset. **(F)** Single-cell analysis showed that PRSS23 and FGF2 were both highly expressed in mesenchymal GC cells. **(G)** FGF2 and PRSS23 were highly co-expressed in normal stomach tissues.

To further validate the role of FGF2 in macrophage infiltration, immune infiltration analysis by two different algorithms was conducted. The results confirmed that FGF2 was positively associated with macrophage infiltration in GC (**Figures 6A, B**). Besides, survival analysis showed that overexpression of FGF2 predicted poor prognosis in GC (**Figures 6C–E**). Single-cell RNA-seq analysis revealed that FGF2 and PRSS23 were predominantly expressed in gastric fibroblasts and highly co-expressed in normal gastric tissue (**Figures 6F, G**). Consistently, both FGF2 and PRSS23 were closely related to EMT signaling and highly co-expressed with biomarkers of CAFs and mesenchymal cells (**Figures S1A-D**). Thus, we speculated that PRSS23 may regulate macrophage infiltration *via* regulating FGF2 secretion in fibroblasts or mesenchymal cells.

**Figure 7:**
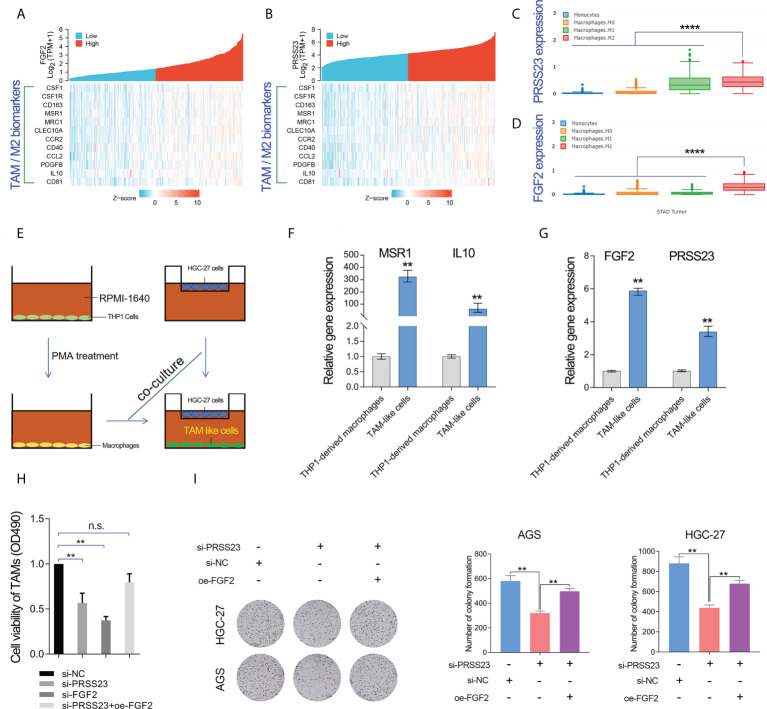
PRSS23 enhances TAM infiltration by regulating FGF2 expression and secretion. **(A)** The gene expression correlation between FGF2 and the well-known biomarker genes of TAM/M2 macrophage was analyzed. **(B)** The gene expression correlation between PRSS23 and the well-known biomarker genes of TAM/M2 macrophage was analyzed. **(C, D)** PRSS23 and FGF2 was upregulated in M2 macrophages. **(E)** The TAM-like cells were induced by co-culturing with HGC-27 cells and THP-1 derived macrophages. **(F)** The well-known biomarkers of TAM/M2 macrophages were greatly upregulated. **(G)** Both FGF2 and PRSS23 were significantly upregulated in TAM-like cells. **(H)** Overexpression of FGF2 rescued the inhibitory effect of survival of TAM-like cells by PRSS23 depletion. **(I)** Overexpression of FGF2 rescued the inhibitory effect of GC cell proliferation by PRSS23 depletion. **p < 0.01, ****p < 0.0001, ns means no significant..

As described above, FGF2 has been shown to play a critical role in TAMs infiltration (39–41). Multiple surface molecules (such as CD163, MSR1 (CD204), MRC1 (CD206), CSF1R, CD40 and CD81) and secreted factors (such as IL10, PDGFB and CCL2) have been reported to be well-known biomarkers of TAM/M2 (20, 42). Hence, we conducted the gene expression correlation analysis between PRSS23/FGF2 and these M2/TAM biomarkers. The results showed that both PRSS23 and FGF2 were highly co-expressed with M2/TAM biomarker genes (**Figures 7A, B**). Besides, we further analyzed the expression level of PRSS23/FGF2 in monocytes and different stages of macrophages. The results showed that both PRSS23 and FGF2 were significantly overexpressed in M2 macrophage, which is highly similar to tumor associated macrophage (**Figures 7C, D**).

Considering high level of secreted FGF2 would have a more pronounced effect in regulating macrophage polarization, we herein selected a GC cell line HGC-27 with relatively high expression of FGF2 for co-culture with THP-1 cells (**Figure 7E**). Then, we examined the expression of popular M2/TAM biomarkers in TAM-like cells by qRT-PCR assay. Both MSR1 (CD206) and IL10 were greatly upregulated in the TAM-like cells, suggested that we successfully induced TAM cells (**Figure 7F**). Consistent with previous immune infiltration analysis, both FGF2 and PRSS23 were significantly upregulated in TAM-like cells (**Figure 7G**). More importantly, knockdown of either PRSS23 or FGF2 significantly reduced the survival rate of TAM-like cells, indicating that both PRSS23 and FGF2 were required for TAM macrophage infiltration (**Figure 7H**). Furthermore, rescue assay confirmed FGF2 overexpression can recovery the inhibitory effect of PRSS23 depletion on cell survival rate of TAM-like cells or cell proliferation of GC cells (**Figures 7H, I**).

**Figure 8:**
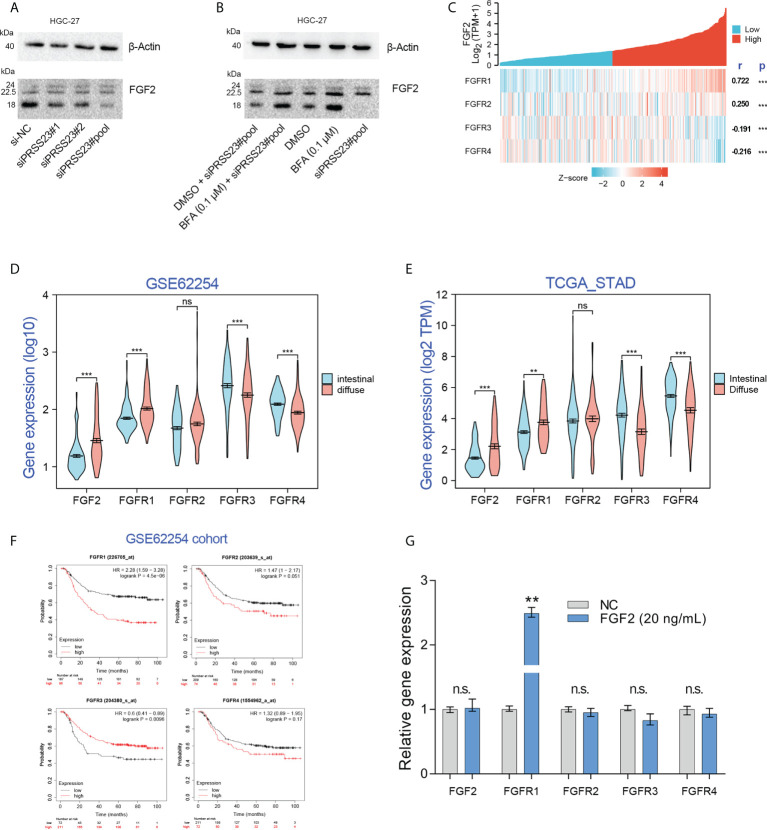
Exogenous recombinant FGF2 significantly upregulated FGFR1 expression in GC. **(A)** Western blotting assay confirmed that PRSS23 knockdown mainly affected low weight molecular FGF2 (18 kDa) expression in GC. **(B)** The experiments combining blockade of FGF2 secretion by Brefeldin A (BFA) with PRSS23 knockdown were performed. **(C)** The gene expression correlation between FGF2 and its receptors in TCGA_STAD cohort. **(D, E)** The expression levels of FGF2/FGFR1/2/3/4 were analyzed in two independent GC cohorts. **(F)** Survival analysis of FGF2/FGFR1/2/3/4 in GSE62254 cohort. **(G)** The expression levels of FGF2/FGFR1/2/3/4 were determined by qRT-PCR after treatment with recombinant FGF2 in HGC-27 cells. **p < 0.01, ***p < 0.001, ns means no significant..

As a serine protease, PRSS23 may play a role in FGF2 processing and secretion by directly cleaving FGF2 proteins. Immunoblotting assay showed that PRSS23 knockdown mainly downregulating 18kDa FGF2 expression (**Figures 8A, B**). However, there is no new FGF2 band generated, even under conditions where Brefeldin A blocked FGF2 secretion (**Figures 8A, B**).

FGF2 has been reported to bind all 4 FGF receptors (FGFR1-4) (43). Gene expression correlation analysis showed that FGF2 expression was positively associated with FGFR1/2, but negatively associated with FGFR3/4 expression (**Figure 8C**). Besides, clinical analysis showed that FGF2 and FGFR1 were highly expressed in diffuse GC, FGFR3 and FGFR4 were lowly expressed in diffuse GC (**Figures 8D, E**). Although there were several studies have reported that FGFR2 was amplified in diffuse GC, our data herein showed that FGFR2 expression has no significant change between diffuse GC and intestinal GC. That may be due to the low frequency (approximately 4-10%) of FGFR2 amplification events in diffuse GC (44–46). Survival analysis showed that FGFR1 overexpression predicted poor prognosis, FGFR3 overexpression predicted favorable prognosis. These results implied that there may be a FGF2/FGFR1 autorinal loop in GC (**Figure 8F**).

Several studies have reported that FGF2 can act in autocrine modes by binding to FGFR1 (47–49). Since FGF2 mRNA and protein level were both downregulated after PRSS23 knockdown, we thus further identified if PRSS23 knockdown downregulated FGF2 mRNA level by affecting FGF2 in an autocrinal manner. In other words, it’s possible that the reduced secreted FGF2 by PRSS23 knockdown may in turn regulate FGF2 transcription *via* an autocrinal loop. Thus, we performed exogenous recombinant FGF2 protein treatment in HGC-27 cells. The results showed that recombinant FGF2 significantly upregulated FGFR1 expression but has no significant effects on FGF2 and FGFR2/3/4 expression in GC (**Figure 8G**).

**Figure 9:**
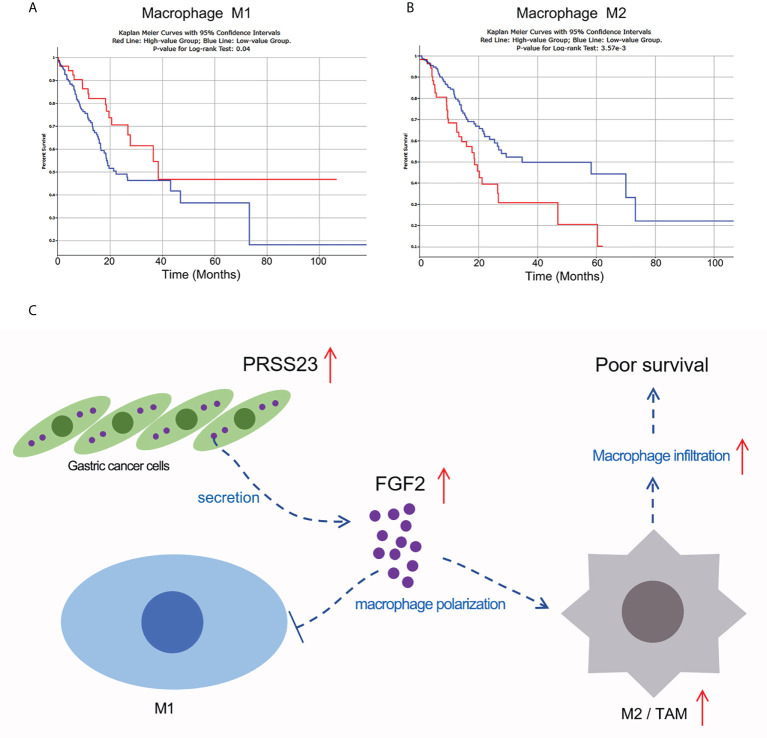
Working model of PRSS23/FGF2 axis in regulating macrophage infiltration. **(A)** High level of M1 macrophage infiltration predicted favorable prognosis in GC. **(B)** High level of M2 macrophage infiltration predicted poor prognosis in GC. **(C)** Working model of PRSS23/FGF2 axis in macrophage infiltration. PRSS23 was overexpressed in GC, which enhanced the expression and secretion of FGF2. Meanwhile, FGF2 upregulation drives macrophage polarized towards M2/TAM phenotype, thereby resulting poor prognosis in GC. Taken together, PRSS23 promotes TAM/M2 macrophage infiltration through positively regulating FGF2 expression and secretion.

Macrophage infiltration can be divided into M1 macrophage infiltration and M2 macrophage infiltration. To this end, we used the quanTIseq algorithm to distinguish M1 macrophages from M2 macrophages (50), and further analyzed the correlation between M1 or M2 macrophage infiltration and the prognosis of GC patients (**Figures 9A, B**). The results showed that GC patients with higher M1 macrophage infiltration tends to possess a longer overall survival time (p=0.04), while GC patients with higher M2 macrophage infiltration tends to possess a shorter overall survival time (p<0.01). Given FGF2 suppressed M1 macrophage polarization but promoted M2 macrophage polarization, we mapped the working model of PRSS23 in promoting GC progression (**Figure 9C**).

In GC, serine protease PRSS23 was overexpressed, thereby promoting the expression and secretion of FGF2. Increased level of FGF2 in turn promotes TAMs polarization and infiltration, leading to poor prognosis in GC. This study reveals for the first time the biological function of PRSS23 in macrophage infiltration, which may have implications for immunotherapy of GC.

## Discussion

Gastric cancer is a common malignancy characterized by significant clinical heterogeneity and remains the fourth most common cause of death resulting from cancer worldwide (51). The intratumor heterogeneity determines the differences in drug resistance, treatment methods and prognosis of different patients. Biomarkers are one of the important ways to distinguish tumor heterogeneity. Therefore, the development of novel biomarker genes is of great significance to the diagnosis, treatment and prognosis of tumors.

In this study, the clinical value of PRSS23 was analyzed in two independent cohorts. PRSS23 overexpression showed a significant correlation with malignant progression and poor prognosis of GC, suggested PRSS23 can be served as an ideal prognostic biomarker for GC. Loss-of-function study had confirmed that PRSS23 functioned oncogenic roles in GC progression, which fits well with another reported evidence that PRSS23 knockdown inhibits gastric tumorigenesis (52).

Previous study had reported Fgf2 was secreted by CAFs in mice (23). Likewise, single-cell analysis also showed that FGF2 was specifically expressed in fibroblasts of human stomach. So, what is the role of FGF2 secreted by fibroblasts? Several studies have reported the critical role of FGF2 in macrophage infiltration and polarization. Knockout of Fgf2 in mice significantly decreased macrophage infiltration (40). Likewise, Im et al. have found that TAMs were polarized towards an inflammatory (M1) phenotype in the Fgf2 knockout mice (24). Similarly, Takase et al. also reported that FGF2/FGFR1 axis was required for TAM infiltration in esophageal cancer (25). These data proved that FGF2 promotes macrophage polarization towards an M2/TAM phenotype. FGFBP1 was reported to be a secreted heparin proteins that reversibly bind FGF1 and FGF2, releasing them from the extracellular matrix and increasing the local levels of free ligand available for receptor binding (53). In other words, FGFBP1 contributes to FGF2 secretion, enhancing its binding to the receptors (FGFR1/2/3/4) (43).

In the present work, a novel role of the serine protease PRSS23 in macrophage infiltration was uncovered in GC. Through high-throughput RNA sequencing, we noted that serine protease PRSS23 was involved into positively regulating FGF2/FGFBP1 expression. Consistently, our subsequent qRT-PCR, western blotting and ELISA assay showed that PRSS23 depletion significantly decreased FGF2 expression and secretion. More importantly, HGC-27 cells and THP-1-derived macrophages co-culture assay further confirmed that PRSS23 promoted TAM infiltration in GC through regulating FGF2 expression and secretion.

Although our findings demonstrate the positive regulation of FGF2 expression and secretion by PRSS23, a non-negligible limitation of our work lies in how exactly PRSS23 regulates FGF2/FGFBP1 expression. Previous studies have reported that although most of FGFs are secreted proteins with cleavable amino terminal portions, FGF1 and FGF2 have no secretion sequences, although they are found in the extracellular compartment (18). In addition, considering that FGF2 mRNA was also decreased by PRSS23 knockdown, this strongly implies that FGF2 was not a direct substrate protein of PRSS23.

Previous publications had reported that ED-71 and its analogues (1, 25-dihydroxyvitamin D3) suppressed expression of FGFBP1/FGF2 by upregulating IκBα (NFKBIA), a critical regulator of NF-κB pathway (54–56). However, according to our RNA-seq data, NFKBIA expression was slightly downregulated in PRSS23-depleted GC cells. The molecular mechanism of how PRSS23 regulates FGF2 expression remains to be further investigated.

TAMs have very similar phenotypes with M2 macrophages, which functioned oncogenic roles in tumor progression (57–59). While M1 macrophages with pro-inflammation functions played tumor-suppressive roles in tumor progression (60). Herein, after differentiation of M1 and M2 macrophages by the quanTIseq algorithm (50), we analyzed the relationship between M1 or M2 macrophage infiltration and the survival of GC patients from TCGA. The results showed that M1 macrophage infiltration predicted favorable prognosis, while M2 macrophage infiltration predicted poor prognosis in GC, suggested M1 and M2 macrophage play opposite roles in GC progression (**Figures 9A, B**). Therefore, we thought PRSS23 plays critical roles in GC progression by enhancing TAMs infiltration *via* FGF2.

## Conclusion

In summary, PRSS23 was overexpressed and showed a significant correlation with poor prognosis, macrophage infiltration. Mechanismly, PRSS23 promotes tumor associated macrophage infiltration by regulating FGF2 expression and secretion. Our finding highlights that PRSS23/FGF2 was a novel signaling axis involved into regulating TAMs infiltration and GC progression.

## Data availability statement

The datasets presented in this study can be found in online repositories. The names of the repository/repositories and accession number(s) can be found in the article/**Supplementary Material**.

## Author contributions

SQ, and DL conceived and designed the study. SQ wrote the paper. DL performed most of the experiments. PH, CH, and ZW carried out initial data analyses and performed partial of the experiments. All authors contributed to drafting the manuscript. All authors have read and approved the final submitted manuscript.

## Funding

This study was supported by grants from the National Natural Science Foundation of China (82203829, 82273451 and 81802375); Hubei Provincial Natural Science Foundation (2022CFB for DL and SQ), the Faculty Development Grants from Hubei University of Medicine (2020QDJZR024 to CH and 2020QDJZR012 to PH) and the Grants of Open-Ended Design Project from Hubei Key Laboratory of Embryonic Stem Cell Research (no. 2021ESOF021).

## Conflict of interest

The authors declare that the research was conducted in the absence of any commercial or financial relationships that could be construed as a potential conflict of interest.

## Publisher’s note

All claims expressed in this article are solely those of the authors and do not necessarily represent those of their affiliated organizations, or those of the publisher, the editors and the reviewers. Any product that may be evaluated in this article, or claim that may be made by its manufacturer, is not guaranteed or endorsed by the publisher.

## Supplementary material

Supplementary Figure 1 Both of PRSS23 and FGF2 were highly co-expressed with biomarkers of mesenchymal cells and cancer-associated fibroblasts in GC. **(A, B)** PRSS23 or FGF2 expression was positively correlated with the expression of mesenchymal biomarkers, but negatively correlated with the expression of epithelial biomarkers in GC. **(C, D)** PRSS23 or FGF2 was highly co-expressed with classic biomarkers of cancer-associated fibroblasts (CAFs) in GC.
