# Supplementary figures and images for: Serine protease PRSS23 drives gastric cancer by enhancing tumor associated macrophage infiltration via FGF2
Source: Front Immunol. 2022 Sep 15;13:955841. doi: 10.3389/fimmu.2022.955841 (PMC9520605; doi:10.3389/fimmu.2022.955841)

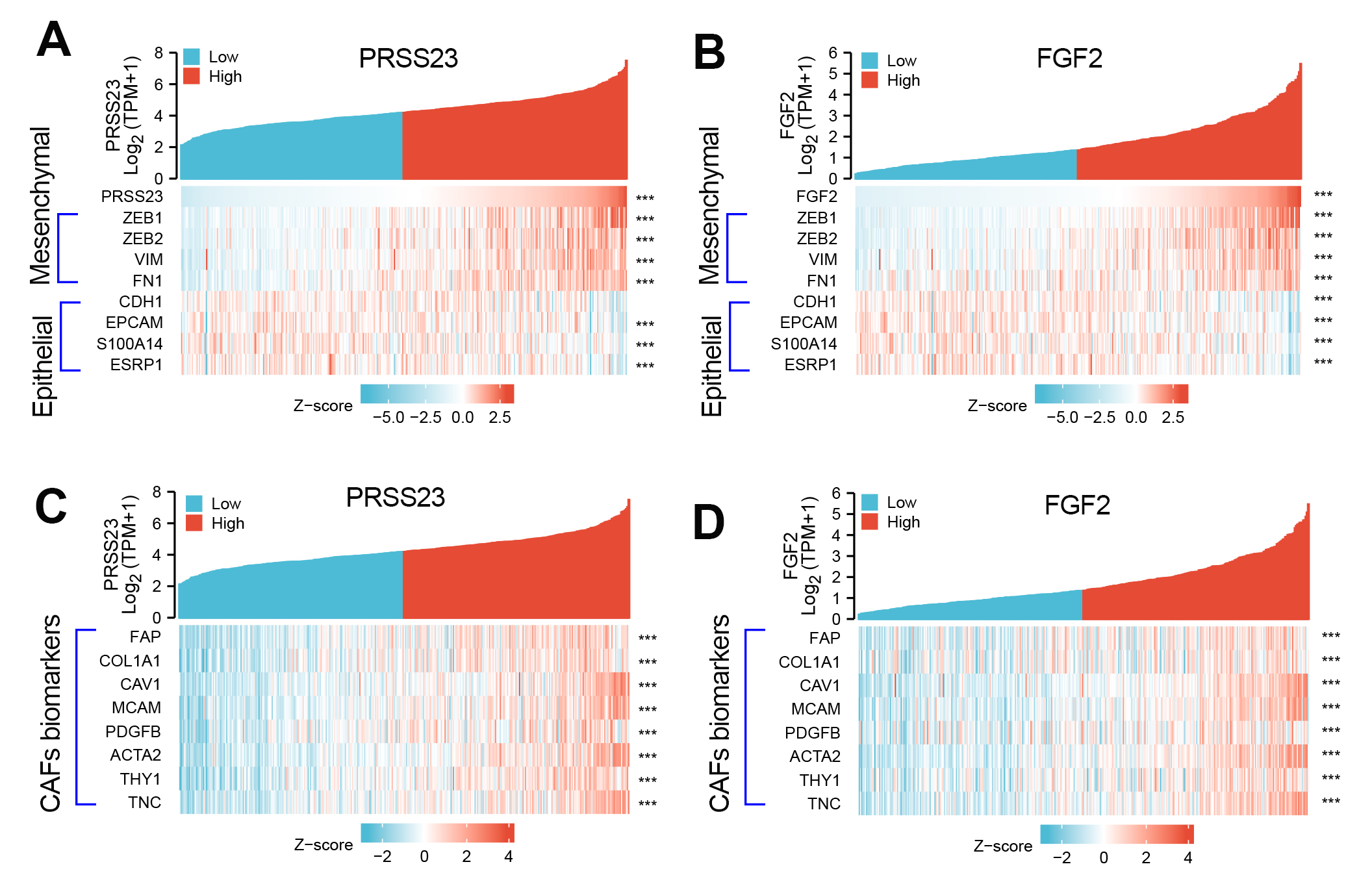

Supplement: Supplementary Figure 1 — Both of PRSS23 and FGF2 were highly co-expressed with biomarkers of mesenchymal cells and cancer-associated fibroblasts in GC. (A, B) PRSS23 or FGF2 expression was positively correlated with the expression of mesenchymal biomarkers, but negatively correlated with the expression of epithelial biomarkers in GC. (C, D) PRSS23 or FGF2 was highly co-expressed with classic biomarkers of cancer-associated fibroblasts (CAFs) in GC. [file Image_1.jpeg]
